# Association of Neutrophil Percentage‐to‐Albumin Ratio (NPAR) With Pathological Features and Long‐Term Prognosis in Diabetic Kidney Disease: A Cohort Study

**DOI:** 10.1096/fj.202601835RRR

**Published:** 2026-09-25

**Authors:** Yang Li, Hongyu Wang, Wendi He, Yi Fang, Shi Jin, Hong Liu, Jie Li, Nana Song, Xiaoqiang Ding, Yan Dai

**Affiliations:** ^1^ Department of Nephrology, Zhongshan Hospital Fudan University Shanghai China; ^2^ Shanghai Key Laboratory of Kidney and Blood Purification Shanghai China; ^3^ Shanghai Medical Center of Kidney Disease Shanghai China; ^4^ Shanghai Institute of Kidney and Dialysis Shanghai China

**Keywords:** diabetic kidney disease, inflammation, neutrophil percentage‐to‐albumin ratio, prognosis, renal biopsy

## Abstract

Growing evidence highlights the central role of inflammation in the onset and progression of DKD. In this study, we investigate the association between the neutrophil percentage‐to‐albumin ratio (NPAR) and clinicopathological features, as well as long‐term renal outcomes, in patients with diabetic kidney disease (DKD). Three hundred five biopsy‐confirmed DKD patients with baseline eGFR ≥ 15 mL/min/1.73 m^2^ were retrospectively enrolled in this study. Baseline clinical and pathological characteristics were compared across NPAR quartiles. The primary outcome was 2‐year DKD progression, defined as a > 40% increase in serum creatinine, end‐stage renal disease, or all‐cause mortality. Multivariable logistic regression, restricted cubic spline (RCS) analysis, and subgroup analyses were conducted to examine the association between the NPAR and kidney outcomes. Subsequently, a prognostic nomogram was constructed for DKD patients incorporating NPAR to stratify individual risk. Higher baseline NPAR quartiles were significantly associated with renal dysfunction and advanced renal pathological lesions. In a subset of renal biopsy specimens, MPO immunostaining demonstrated increased renal neutrophil infiltration in patients with higher NPAR quartiles. Renal MPO expression was positively correlated with mesangial expansion, interstitial fibrosis, and tubular atrophy. Among patients with complete follow‐up, 65.3% developed the composite kidney outcome. Compared with the lowest NPAR quartile, multivariable‐adjusted odds ratios (95% CIs) for kidney outcomes were 1.68 (0.36–8.15), 6.21 (1.35–33.55), and 6.88 (1.27–46.86) for Q2, Q3, and Q4, respectively. RCS analysis indicated a nonlinear association between NPAR and kidney outcomes, with risk increasing sharply above an NPAR value of 1.38. Subgroup analyses identified significant interactions between NPAR and Kimmelstiel–Wilson nodules as well as SGLT2 inhibitor use on kidney outcomes. A prognostic model incorporating NPAR quartiles, age, total cholesterol, eGFR, and interstitial fibrosis demonstrated good discrimination (AUROC 0.863). In conclusion, elevated NPAR is associated with increased renal neutrophil infiltration, more severe histopathological injury, and adverse renal outcomes in patients with DKD, and independently predicts long‐term renal progression.

## Graphical abstract

This graphical abstract summarizes the association between the neutrophil percentage‐to‐albumin ratio (NPAR), renal pathology, and prognosis in biopsy‐confirmed diabetic kidney disease. Higher NPAR was associated with increased renal MPO‐positive neutrophil infiltration, more severe histopathological injury, and poorer renal outcomes. Incorporation of NPAR into a prognostic model improved individualized risk stratification, highlighting its potential as a simple, readily available biomarker for clinical risk assessment.

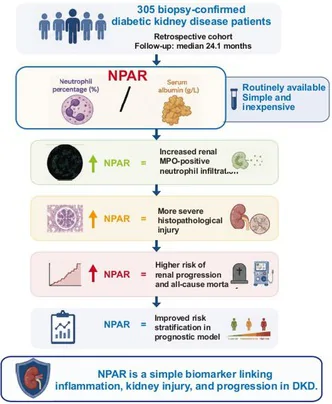

## 1 Introduction

Diabetic kidney disease (DKD) is one of the major microvascular complications of diabetes and progresses to end‐stage renal disease (ESRD) in approximately 40% of affected individuals [1, 2]. With more than 118 million people living with diabetes, China has witnessed DKD surpass glomerulonephritis as the leading cause of chronic kidney disease (CKD) [3]. Patients with DKD face a substantial risk of progression to ESRD, contributing significantly to morbidity and mortality. Early identification of individuals at high risk for rapid disease progression is essential for timely intervention and improved prognosis.

Growing epidemiological and experimental evidence highlights the central role of inflammation in the onset and progression of DKD [4]. Clinical biopsies reveal that both circulating inflammatory mediators and infiltration of immune cells levels into renal tissue have been elevated in patients with DKD, with upregulation of adhesion molecules and chemokine, being directly associated with the prototypical clinical marker albuminuria and progressive eGFR decline [5, 6, 7]. The neutrophil percentage‐to‐albumin ratio (NPAR) is a recently proposed biomarker that integrates systemic inflammation and nutritional status. As an easily obtainable and inexpensive index, NPAR has been associated with all‐cause mortality in acute kidney injury (AKI) [8], increased risk of CKD [9], metabolic syndrome (MetS) [10], diabetic retinopathy [11], and the prevalence of DKD in Type 2 diabetes [12]. However, previous studies often relied on clinical diagnoses without biopsy confirmation, raising concerns about misclassification with non‐diabetic renal diseases.

Renal biopsy remains the gold standard for diagnosing DKD, with characteristic pathological features such as Kimmelstiel–Wilson nodules. Despite the recognized value of NPAR as an inflammatory marker [13], its relationship with the clinical presentation, pathological severity, and long‐term prognosis of biopsy‐confirmed DKD has not been fully elucidated. Therefore, in this retrospective cohort study, we enrolled biopsy‐confirmed DKD patients and followed them for 2 years to investigate the association between NPAR levels and clinicopathological features, as well as renal outcomes.

## 2 Materials and Methods

### 2.1 Patients and Study Design

This retrospective cohort study was conducted at Zhongshan Hospital, Fudan University. A total of 348 patients aged 18–80 years with Type 1 or Type 2 diabetes who underwent renal biopsy between January 2018 and October 2023 were screened. Patients were included if they had biopsy‐confirmed DKD as the sole renal diagnosis. Individuals with baseline CKD Stage 5 (eGFR < 15 mL/min/1.73 m^2^) were excluded. A total of 305 eligible DKD patients were enrolled at baseline. After excluding patients who were lost to follow‐up and those who developed AKI attributable to causes other than DKD during follow‐up, 141 patients remained with complete 2‐year renal outcome data for analysis. Baseline demographic, clinical, and pathological data were collected at the time of biopsy. Patients were followed for 2 years to assess renal outcomes. This study was approved by the Clinical Research Ethics Committee of Zhongshan Hospital (Approval No.: B2023‐076R) and conducted in accordance with the Declaration of Helsinki. Written informed consent was obtained from all participants prior to renal biopsy.

### 2.2 Diagnostic Criteria for DKD

**FIGURE 1:**
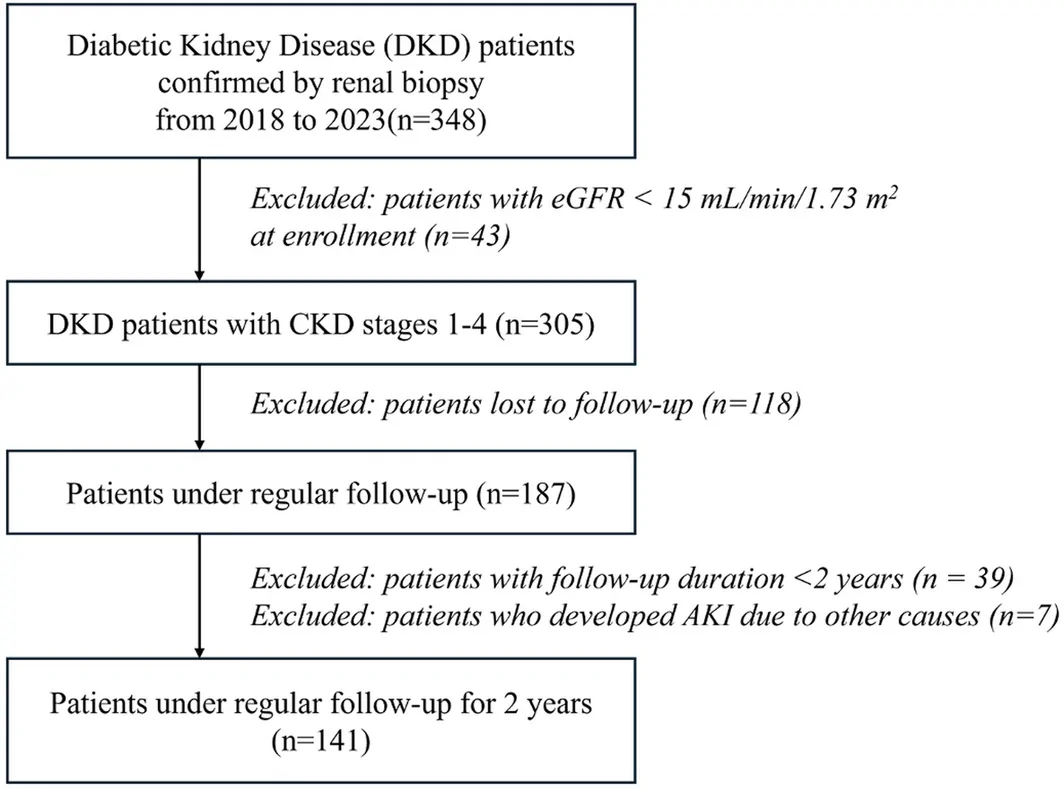
Flowchart of participant selection.

Renal biopsy was performed when non‐diabetic renal disease (NDRD) was clinically suspected, such as unexplained hematuria, proteinuria without diabetic retinopathy, or rapid deterioration of renal function. All biopsy specimens were processed at Zhongshan Hospital. DKD diagnosis was based on light microscopy and immunofluorescence findings. A minimum of eight glomeruli per biopsy was required. Pathological scoring followed the criteria of Mottl et al. [14]. The definitions of pathologic scoring are summarized in Figure 1. Evaluation of kidney histologic lesions was graded according to Renal Pathology Society (RPS) Diabetic Nephropathy Classification [15] with the following classes of glomerular lesions: (i) Class I was glomerular basement thickening (GBM > 395 nm in females and > 430 nm in males) and only mild, nonspecific changes on light microscopy; (ii) Class II was mild (IIa) or severe (IIb) mesangial expansion in > 25% of the observed mesangium without either nodular lesions or global sclerosis in > 50% of the glomeruli; (iii) Class III was nodular lesions without global sclerosis in > 50% of the glomeruli (presence of at least one convincing Kimmelstiel–Wilson nodule); and (iv) Class IV was global sclerosis in > 50% of the glomeruli. Two experienced renal pathologists blinded to patient outcomes reviewed all kidney biopsy slides.

### 2.3 Clinical Data Collection

Patient demographic and physical examination data—including age, gender, body mass index (BMI), history of hypertension, and medical history—were collected using a standardized case report form. Clinical biochemical parameters, measured from the most recent tests prior to kidney biopsy, comprised neutrophil percentage, albumin (Alb), hemoglobin (Hb), C‐reactive protein (CRP), fasting blood glucose (FBG), glycated hemoglobin (HbA1c), serum creatinine (SCr), cystatin C (CysC), blood urea nitrogen (BUN), uric acid (UA), total cholesterol, low‐density lipoprotein cholesterol (LDL), high‐density lipoprotein cholesterol (HDL), triglycerides, 24‐h proteinuria, and urinary albumin‐creatinine ratio (UACR).

Treatment information, including use of renin‐angiotensin system (RAS) blockers, sodium‐glucose co‐transporter 2 inhibitors (SGLT2i), and lipid‐lowering statins, was obtained from medical records at the time of kidney biopsy. NPAR was calculated as follows: neutrophil percentage (%) / albumin (g/dL). Renal function was assessed by estimating the glomerular filtration rate (eGFR) using the creatinine‐based CKD‐EPI equation [16].

Following baseline assessment, patients were scheduled for regular follow‐up visits every 3 months to monitor changes in key indicators such as eGFR and UACR.

### 2.4 Renal Outcomes

Follow‐up outcomes were retrospectively ascertained from available medical records, with the final follow‐up date set at October 31, 2025. The renal outcome was defined as the DKD progression, which was determined by the occurrence of any of the following events within 2 years: (1) an increase in serum creatinine of > 40%; (2) the development of end‐stage renal disease (ESRD), defined as the initiation of hemodialysis, peritoneal dialysis, or kidney transplantation; or (3) all‐cause mortality. A confirmatory second serum creatinine measurement was not required for the definition of the > 40% increase endpoint. To minimize potential misclassification, all cases meeting this criterion were retrospectively reviewed, and events attributable to acute kidney injury (AKI) were excluded according to the predefined exclusion criteria.

### 2.5 Immunofluorescence (IF) Staining

Kidney biopsy specimens were fixed, paraffin‐embedded, and sectioned at a thickness of 3 μm. Adjacent sections were stained with hematoxylin and eosin (H&E) using standard procedures. For immunofluorescence staining, paraffin‐embedded kidney sections were deparaffinized and subjected to antigen retrieval in sodium citrate buffer. After blocking with 5% sheep serum for 30 min at 37°C, the sections were incubated overnight at 4°C with an anti‐MPO primary antibody (1:200; Proteintech, #22225‐1‐AP). The sections were then incubated with an Alexa Fluor 488‐conjugated secondary antibody (anti‐rabbit, AF488A18740) for 2 h at room temperature, followed by three washes with PBS. Nuclei were counterstained with DAPI. Images were acquired under identical microscope settings for all specimens. MPO immunofluorescence was quantified using ImageJ software (National Institutes of Health, Bethesda, MD, USA). The mean fluorescence intensity (MFI) of MPO staining was measured in five randomly selected non‐overlapping high‐power fields (×200) for each specimen, and the average MFI for each specimen was used for statistical analysis.

### 2.6 Statistical Analysis

Continuous variables with normal distributions were reported as mean and standard deviation (SD), whereas those with non‐normal distributions were reported as median and interquartile range (IQR). Categorical variables were summarized as frequencies and percentages. Linear trend tests were used to assess monotonic trends across NPAR quartiles for normally distributed continuous outcomes, whereas the Jonckheere–Terpstra test was used for non‐normally distributed continuous outcomes. For categorical outcomes, trend analysis across NPAR quartiles was conducted using the Cochran–Mantel–Haenszel (CMH) test, with stratification applied where appropriate to account for potential confounding.

Multivariable logistic regression was performed to evaluate the associations between NPAR levels—categorized both as quartiles and as an ordinal variable—and the 2‐year composite kidney outcome. Four models were constructed: Model 1 adjusted for age, sex, BMI, and hypertension history; Model 2 further adjusted for serum uric acid, estimated glomerular filtration rate (eGFR), fasting blood glucose, total cholesterol (TC), C‐reactive protein (CRP), and DKD grade; Model 3 additionally included Kimmelstiel–Wilson (K–W) nodule presence, renal interstitial fibrosis, and renal tubular atrophy; and Model 4 further incorporated lipid‐lowering treatment and ACEI/ARB use. Subgroup analyses were conducted stratifying by age (< 60 vs. ≥ 60 years), sex (male vs. female), TC (< 6.2 vs. ≥ 6.2 mmol/L), eGFR (< 60 vs. ≥ 60 mL/min/1.73 m^2^), DKD grade (1–2 vs. 3–4), K–W nodule presence (yes vs. no), ACEI/ARB use (yes vs. no), SGLT2 inhibitor (SGLT2i) use (yes vs. no), and lipid‐lowering treatment (yes vs. no). Likelihood ratio tests were used to formally assess effect modification (i.e., statistical interactions) across these subgroups. Furthermore, we conducted a sensitivity analysis to compare baseline characteristics between DKD patients with and without follow‐up. To address potential confounding attributable to differential loss to follow‐up, we applied inverse probability of treatment weighting (IPTW) in Model 4 to balance baseline covariates; the association between NPAR and 2‐year DKD progression was subsequently re‐estimated in the IPTW‐weighted sample.

Dose–response relationships between NPAR and the 2‐year composite kidney outcome were assessed using a restricted cubic spline (RCS) model with three internal knots. A statistically significant nonlinearity test justified modeling the association flexibly. Based on the fitted RCS curve, the NPAR value at which the estimated OR was closest to 1 was identified as a risk‐transition point. This value was used to aid interpretation of the nonlinear exposure‐response relationship between NPAR and 2‐year DKD progression. To further explore effect heterogeneity by age and kidney function in the association between NPAR and DKD prognosis, subgroup analyses were conducted stratifying patients by age (< 60 vs. ≥ 60 years) and estimated glomerular filtration rate (eGFR) (< 60 vs. ≥ 60 mL/min/1.73 m^2^).

During model development, the group penalized regression was first applied to select candidate predictors with non‐zero coefficients. The group penalized estimator was defined as: ${\hat{\beta }}_{\lambda }=\arg_{\beta }\min\left({\left|\left|Y-\mathrm{X\beta}\right|\right|}_{2}^{2}+\lambda \sum_{g=1}^{G}{\left\|\beta_{l_{g}}\right\|}_{2}\right)$, where β represents the regression coefficients, *λ* is the tuning parameter controlling the degree of shrinkage. Ten‐fold cross‐validation is used to choose the minimal λ value. The penalty of $l_{g}$ is the index set of *g*th grouping variables. It makes variable select at group levels and keep invariant under orthogonal transformations. The group minimax concave penalty (grMCP) was applied for group selection. Subsequently, a nomogram was constructed using these selected variables. Model performance was evaluated using the area under the receiver operating characteristic curve (AUROC), calibration plots, and decision curve analysis (DCA). To facilitate clinical interpretation, patients were stratified into low‐, intermediate‐, and high‐risk groups using two cut‐off values derived from the Youden index. The cumulative incidence of DKD progression and corresponding relative risks (RRs) were then estimated for each risk group.

All statistical tests were two‐tailed, and differences with *p* < 0.05 were considered statistically significant. All statistical analyses were conducted using the packages in R software version 4.2.1 (R core team).

## 3 Results

### 3.1 Participants Characteristics

Figure 1 shows the flowchart of participant inclusion. Initially, 348 patients with renal biopsy‐confirmed DKD were recruited. After excluding those with baseline eGFR < 15 mL/min/1.73m^2^, 305 eligible patients were included in the final analysis. The cohort had a mean age of 55.22 ± 11.60 years; 81.0% were male, and 94.1% had hypertension. The mean NPAR value was 1.97 ± 0.62, which was subsequently stratified into four quartiles: Q1 (< 1.55), Q2 (1.55–1.86), Q3 (1.86–2.27), and Q4 (≥ 2.27). Higher NPAR quartiles were associated with significantly lower hemoglobin and lymphocyte counts, together with elevated levels of total cholesterol, CRP, erythrocyte sedimentation rate, and procalcitonin. Additionally, renal function showed progressive impairment across increasing NPAR quartiles, manifested by a decrease in eGFR along with elevated levels of blood urea nitrogen, serum uric acid, serum creatinine, urinary protein, UACR, urinary β2‐microglobulin, urinary α1‐microglobulin, and urinary transferrin (Table 1). Table 2 presents the renal pathological and therapeutic characteristics across NPAR quartiles. Patients in higher NPAR quartiles were more likely to have advanced diabetic nephropathy class, K–W nodules and exudative lesions; the proportion of renal interstitial fibrosis and tubular atrophy also increased significantly with higher NPAR quartiles. Furthermore, patients in higher NPAR quartiles had a lower probability of receiving ACEI/ARB treatment.

**TABLE 1:** Demographic and clinical characteristics of patients with DKD across various NPAR quartiles.

Variable	NPAR				Statistics	*p*
	Q1 (< 1.55)	Q2 (1.55–1.86)	Q3 (1.86–2.27)	Q4 (≥ 2.27)		
Age, years	55.18 ± 13.04	56.96 ± 10.17	54.74 ± 11.41	54.01 ± 11.60	−0.960	0.338^a^
Male, *n* (%)	68 (88.3)	60 (78.9)	55 (72.4)	64 (84.2)	0.899	0.343
BMI, kg/m^2^	25.40 ± 3.22	24.68 ± 3.54	25.27 ± 3.93	25.39 ± 3.51	0.291	0.771^a^
Smoking, *n* (%)	28 (36.4)	27 (35.5)	21 (27.6)	26 (34.2)	1.216	0.270^b^
Alcohol use, *n* (%)	17 (22.1)	17 (22.1)	10 (13.2)	11 (14.5)	2.639	0.104^b^
Hypertension, *n* (%)	72 (93.5)	75 (98.7)	71 (93.4)	69 (90.8)	4.488	0.213^b^
Diabetes type, *n* (%)					< 0.001	0.992^b^
T1DM	2 (2.6)	2 (2.6)	3 (3.9)	3 (3.9)		
T2DM	75 (97.4)	74 (97.4)	72 (94.7)	72 (94.7)		
Other types	0 (0)	0 (0)	1 (1.3)	1 (1.3)		
Diabetes duration, years	11.79 ± 7.36	11.63 ± 6.34	11.46 ± 6.31	12.32 ± 6.42	0.412	0.681^a^
Hb, g/L	123.99 ± 20.44	112.22 ± 21.48	104.53 ± 21.48	101.13 ± 19.42	−7.190	< 0.001^a^
NC, ×10^9^/L	3.56 ± 1.10	3.89 ± 1.41	4.63 ± 3.07	5.56 ± 2.93	5.749	< 0.001^a^
NP, %	53.87 ± 7.01	61.63 ± 8.04	65.23 ± 7.00	70.60 ± 7.98	13.940	< 0.001^a^
LC, ×10^9^/L	2.20 ± 0.71	1.66 ± 0.68	1.51 ± 0.55	1.45 ± 0.56	−7.390	< 0.001^a^
MC, ×10^9^/L	0.53 ± 0.17	0.50 ± 0.19	0.55 ± 0.53	0.50 ± 0.21	−0.188	0.851^a^
TP, g/L	65.51 ± 5.59	62.21 ± 6.53	57.21 ± 7.41	49.87 ± 6.92	−15.130	< 0.001^a^
Alb, g/L	40.32 ± 3.53	36.34 ± 4.52	32.26 ± 4.06	25.67 ± 4.49	−22.270	< 0.001^a^
BUN, mmol/L	8.4 [6.8, 10.3]	9.4 [7.5, 11.6]	11.1 [8.1, 14.3]	11.6 [8.0, 15.0]	21 373	< 0.001^c^
SUA, μmol/L	407 [345, 475]	394 [338, 459]	378 [344, 437]	381 [333, 444]	15 818	0.078^c^
SCr, μmol/L	118 [91, 161]	135 [99, 186]	176 [110, 217]	158 [116, 247]	20 781	< 0.001^c^
eGFR, mL/min/1.73 m^2^	62 [42, 80]	47 [30, 63]	35 [24, 63]	39 [25, 58]	13 816	< 0.001^c^
Glu, mmol/L	6.46 ± 2.32	7.50 ± 3.70	6.94 ± 2.90	6.87 ± 4.16	0.385	0.700^a^
HbA1c, %	6.7 [6.2, 8.0]	7.3 [6.3, 8.5]	6.9 [6.1, 8.2]	7.0 [6.2, 8.7]	15 730	0.606^c^
TC, mmol/L	4.42 ± 1.37	4.64 ± 1.50	4.58 ± 1.31	5.85 ± 2.25	4.805	< 0.001^a^
TG, mmol/L	2.21 ± 2.63	2.40 ± 2.19	1.80 ± 1.07	1.94 ± 1.63	−1.359	0.175^a^
LDL‐C, mmol/L	2.72 ± 2.49	2.60 ± 1.21	2.61 ± 1.10	3.80 ± 1.81	3.495	< 0.001^a^
HDL‐C, mmol/L	0.99 ± 0.27	1.06 ± 0.48	1.19 ± 0.43	1.19 ± 0.42	3.538	< 0.001^a^
CRP, mg/L	0.90 [0.50, 1.85]	1.10 [0.50, 2.95]	1.30 [0.50, 3.20]	2.30 [0.80, 9.60]	19 951	< 0.001^c^
ESR, mm/h	16.0 [7.5, 35.0]	31.5 [19.0, 50.5]	40.0 [21.0, 54.5]	55.0 [32.0, 74.5]	16 926	< 0.001^c^
PCT, ng/mL	0.06 [0.04, 0.09]	0.06 [0.05, 0.11]	0.08 [0.05, 0.12]	0.09 [0.06, 0.14]	18 348	< 0.001^c^
24‐h UTP, g/24 h	1.11 [0.62, 2.84]	3.42 [1.75, 4.91]	4.55 [2.07, 6.77]	6.75 [4.32, 10.11]	26 393	< 0.001^c^
UACR, μg/mg Cr	760 [327, 2096]	2110 [1282, 3255]	3460 [1445, 4670]	5519 [3897, 7145]	26 531	< 0.001^c^
uIgG, mg/L	42.5 [13.0, 139.1]	108.2 [54.8, 207.6]	208.2 [90.8, 374.4]	324.4 [232.5, 548.3]	22 493	< 0.001^c^
uβ2‐MG, mg/L	0.75 [0.16, 3.28]	2.21 [0.87, 5.97]	3.16 [0.63, 7.68]	5.84 [1.66, 16.83]	19 141	< 0.001^c^
uα1‐MG, mg/L	15.2 [9.8, 25.9]	24.3 [16.3, 37.1]	32.0 [18.4, 49.6]	42.8 [24.3, 62.8]	20 581	< 0.001^c^
uTRF, mg/L	24.2 [7.3, 57.0]	65.2 [37.1, 99.8]	94.7 [52.4, 138.5]	145.6 [102.1, 260.3]	22 903	< 0.001^c^

Abbreviations: 24‐h UTP, 24‐h urinary protein; Alb, albumin; BMI, body mass index; BUN, blood urea nitrogen; CRP, C‐reactive protein; DKD, diabetic kidney disease; eGFR, estimated glomerular filtration rate; ESR, erythrocyte sedimentation rate; Glu, glucose; Hb, hemoglobin; HbA1c, glycated hemoglobin; HDL‐C, high‐density lipoprotein cholesterol; LC, lymphocyte count; LDL‐C, low‐density lipoprotein cholesterol; MC, monocyte count; NC, neutrophil count; NP, neutrophil percentage; NPAR, neutrophil percentage‐to‐albumin ratio; PCT, procalcitonin; SCr, serum creatinine; SUA, serum uric acid; T1DM, Type 1 diabetes mellitus; T2DM, Type 2 diabetes mellitus; TC, total cholesterol; TG, triglycerides; TP, total protein; UACR, urinary albumin‐to‐creatinine ratio; uIgG, urinary immunoglobulin G; uTRF, urinary transferrin; uα1‐MG, urinary α1‐microglobulin; uβ2‐MG, urinary β2‐microglobulin.

^a^ Linear trend tests.

^b^ Cochran–Mantel–Haenszel (CMH) test.

^c^ Jonckheere–Terpstra test.

**TABLE 2:** Renal pathological and therapeutic characteristics of patients with DKD across various NPAR quartiles.

Variable	NPAR				Statistics	*p*
	Q1 (< 1.55)	Q2 (1.55–1.86)	Q3 (1.86–2.27)	Q4 (≥ 2.27)		
Diabetic nephropathy class, *n* (%)					16.423	< 0.001^a^
Class 1–2	30 (39.0)	11 (14.5)	8 (10.5)	4 (5.3)		
Class 3	28 (36.4)	36 (47.4)	43 (56.6)	42 (55.3)		
Class 4	19 (24.7)	29 (38.2)	25 (32.9)	30 (39.5)		
Glomerular lesion grade, *n* (%)					0.106	0.745^a^
Grades 1–3	4 (5.2)	1 (1.3)	2 (2.6)	6 (7.9)		
Grade 4	54 (70.1)	46 (60.5)	47 (61.8)	46 (60.5)		
Grade 5	19 (24.7)	29 (38.2)	27 (35.5)	24 (31.6)		
K–W nodule, *n* (%)	39 (50.6)	53 (69.7)	62 (81.6)	68 (89.5)	31.726	< 0.001^a^
Exudative lesion, *n* (%)	14 (18.2)	24 (31.6)	25 (32.9)	35 (46.1)	12.616	< 0.001^a^
Global glomerulosclerosis, *n* (%)					0.290	0.590^a^
Grade 1	39 (50.6)	26 (34.2)	29 (38.2)	34 (44.7)		
Grade 2	20 (26.0)	23 (30.3)	23 (30.3)	21 (27.6)		
Grade 3	18 (23.4)	27 (35.5)	24 (31.6)	21 (27.6)		
Renal interstitial fibrosis, *n* (%)					13.149	< 0.001^a^
Grade 0	13 (16.9)	7 (9.2)	7 (9.2)	3 (3.9)		
Grade 1	50 (64.9)	50 (65.8)	41 (53.9)	45 (57.9)		
Grades 2–3	14 (18.2)	19 (25.0)	28 (36.8)	29 (38.2)		
Renal tubular atrophy, *n* (%)					22.593	< 0.001^a^
Grade 1	36 (46.8)	17 (22.4)	17 (22.4)	13 (17.1)		
Grade 2	26 (33.8)	38 (50.0)	28 (36.8)	26 (34.2)		
Grade 3	15 (19.5)	21 (27.6)	31 (40.8)	37 (48.7)		
Arteriolar hyalinosis, *n* (%)	77 (100.0)	74 (97.4)	73 (96.1)	73 (96.1)	2.593	0.107^a^
Lipid‐lowering treatment, *n* (%)	37 (48.1)	46 (60.5)	49 (64.5)	47 (61.8)	3.249	0.071^a^
SGLT2i treatment, *n* (%)					1.497	0.221^a^
After biopsy	22 (28.6)	15 (19.7)	11 (14.5)	16 (21.1)		
Before biopsy	7 (9.1)	7 (9.2)	8 (10.5)	5 (6.6)		
No	48 (62.3)	54 (71.1)	57 (75.0)	55 (72.4)		
ACEI/ARB treatment, *n* (%)	59 (76.6)	51 (67.1)	49 (64.5)	32 (42.1)	18.328	< 0.001^a^

Abbreviations: ACEI, angiotensin‐converting enzyme inhibitor; ARB, angiotensin receptor blocker; DKD, diabetic kidney disease; K–W nodule, Kimmelstiel–Wilson nodule; NPAR, neutrophil percentage‐to‐albumin ratio; SGLT2i, sodium‐glucose cotransporter 2 inhibitor.

^a^ Cochran–Mantel–Haenszel (CMH) test.

### 3.2 Association Between NPAR and 2‐Year Progression of DKD

Of the initial 305 DKD patients, 187 maintained regular follow‐ups. After excluding patients lost to follow‐up and those who developed AKI from non‐DKD causes, 141 patients had complete 2‐year renal outcome data available for analysis. The composite kidney outcome occurred in 92 patients (65.3%), consisting of progression to ESRD (*n* = 73), death (*n* = 6), and a ≥ 40% increase in serum creatinine (*n* = 13). The association between NPAR and disease progression is summarized in Table 3. Compared with patients in the lowest NPAR quartile (Q1), those in higher quartiles had an increased risk of composite kidney outcome. The crude odds ratios (ORs) with 95% confidence intervals (CIs) were 1.61 (0.55–4.85) for Q2, 4.94 (1.65–16.23) for Q3, and 8.03 (2.25–35.21) for Q4 (Model 1). After multivariable adjustment for demographic, clinical, pathological, and therapeutic factors, this positive association persisted (Model 4), with adjusted ORs (95% CIs) of 1.68 (0.36–8.15), 6.21 (1.35–33.55), and 6.88 (1.27–46.86) for Q2‐Q4, respectively. Furthermore, when NPAR quartiles were treated as an ordinal variable, a significant trend of increasing risk across ascending quartiles was confirmed in Model 4 (OR per quartile increase: 1.92, 95% CI: 1.13–3.48).

**TABLE 3:** Association between NPAR and the 2‐year progression of DKD.

	Model 1	Model 2	Model 3	Model 4
	OR (95% CI)	OR (95% CI)	OR (95% CI)	OR (95% CI)
NPAR				
Q1	Reference	Reference	Reference	Reference
Q2	1.61 (0.55–4.85)	1.15 (0.30–4.549)	1.22 (0.28–5.56)	1.68 (0.36–8.15)
Q3	4.94 (1.65–16.23)	6.12 (1.60–27.26)	4.52 (1.06–22.32)	6.21 (1.35–33.55)
Q4	8.03 (2.25–35.21)	6.44 (1.48–33.58)	6.38 (1.28–39.09)	6.88 (1.27–46.86)
NPAR				
Ordinal	2.21 (1.51–3.38)	2.10 (1.31–3.53)	1.99 (1.20–3.48)	1.92 (1.13–3.48)

*Note:* Model 1 comprised age, gender, BMI, hypertension history; Model 2 included the factors of model 1 and serum uric acid, estimated glomerular filtration rate, fasting blood glucose, total cholesterol, C‐reactive protein, diabetic nephropathy grade; Model 3 included the factors of model 2 and K–W nodule, renal interstitial fibrosis and renal tubular atrophy; Model 4 included the factors of model 3 and lipid‐lowering treatment, SGLT2i and ACEI/ARB Treatment.

Abbreviations: CI, confidence interval; DKD, diabetic kidney disease; NPAR, neutrophil percentage‐to‐albumin ratio; OR, odds ratio.

### 3.3 Subgroup Analysis

We evaluated demographic, clinical, pathological, and treatment‐related factors in relation to the 2‐year composite kidney outcome (Tables S1 and S2). Variables that showed statistical significance were selected as potential effect modifiers. Subsequent subgroup analyses were performed to assess whether these factors modified the association between NPAR and progression risk (Table 4). When stratified by age, sex, eGFR, and lipid‐lowering treatment, the positive association remained consistent, as none of the interaction terms reached statistical significance (all *p* for interaction > 0.05). Notably, significant effect modifications were identified for DKD pathological features and SGLT2i treatments: the association between NPAR and progression risk was stronger in patients with K–W nodule (*p* for interaction = 0.016) and in patients receiving SGLT2i treatment (*p* for interaction = 0.005).

**TABLE 4:**
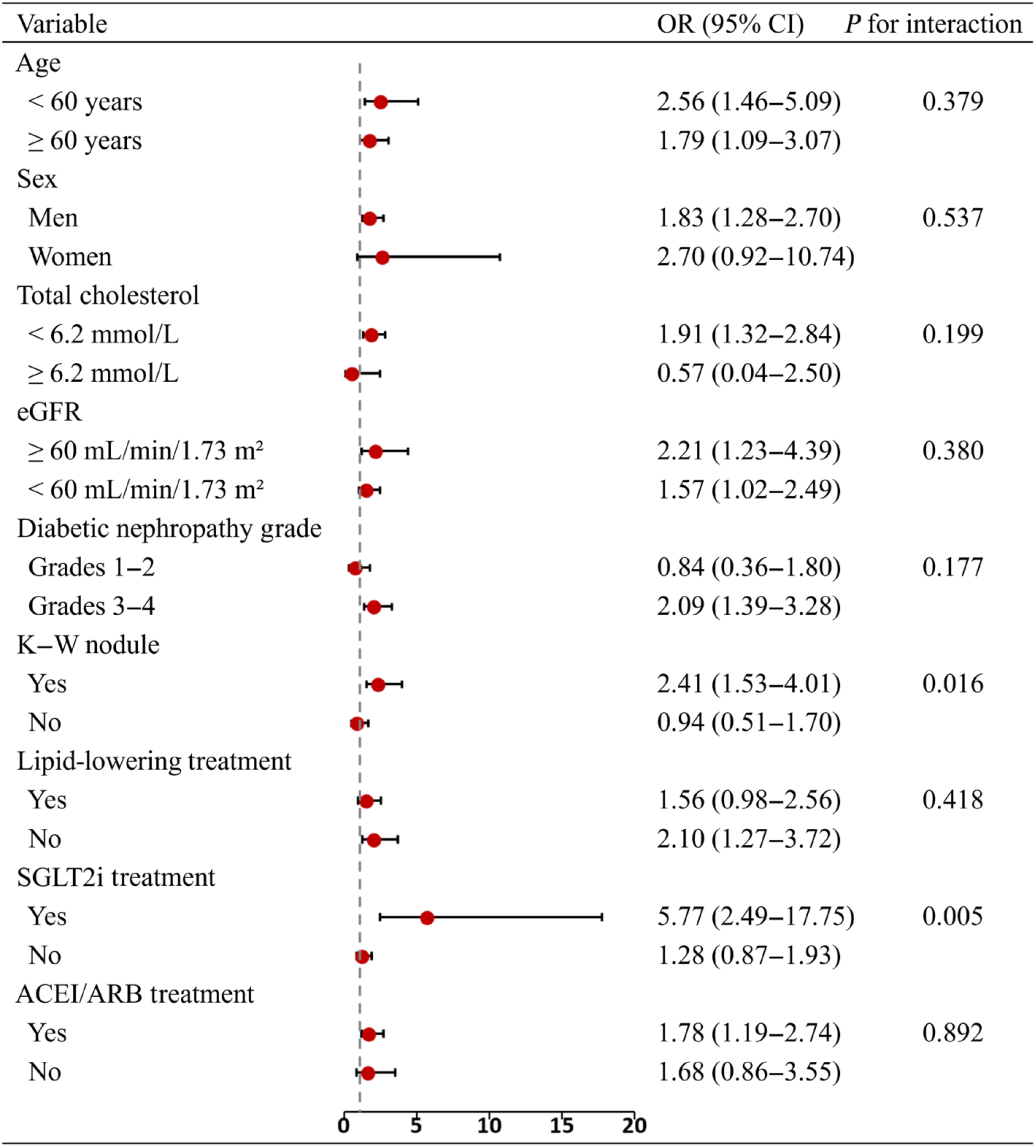
Subgroup analysis of associations between NPAR and the 2‐year progression of DKD.

Abbreviations: ACEI, angiotensin‐converting enzyme inhibitor; ARB, angiotensin receptor blocker; CI, confidence interval; DKD, diabetic kidney disease; eGFR, estimated glomerular filtration rate; K–W nodule, Kimmelstiel–Wilson nodule; NPAR, neutrophil percentage‐to‐albumin ratio; OR, odds ratio; SGLT2i, sodium‐glucose cotransporter 2 inhibitor.

### 3.4 RCS and Threshold Analysis

**FIGURE 2:**
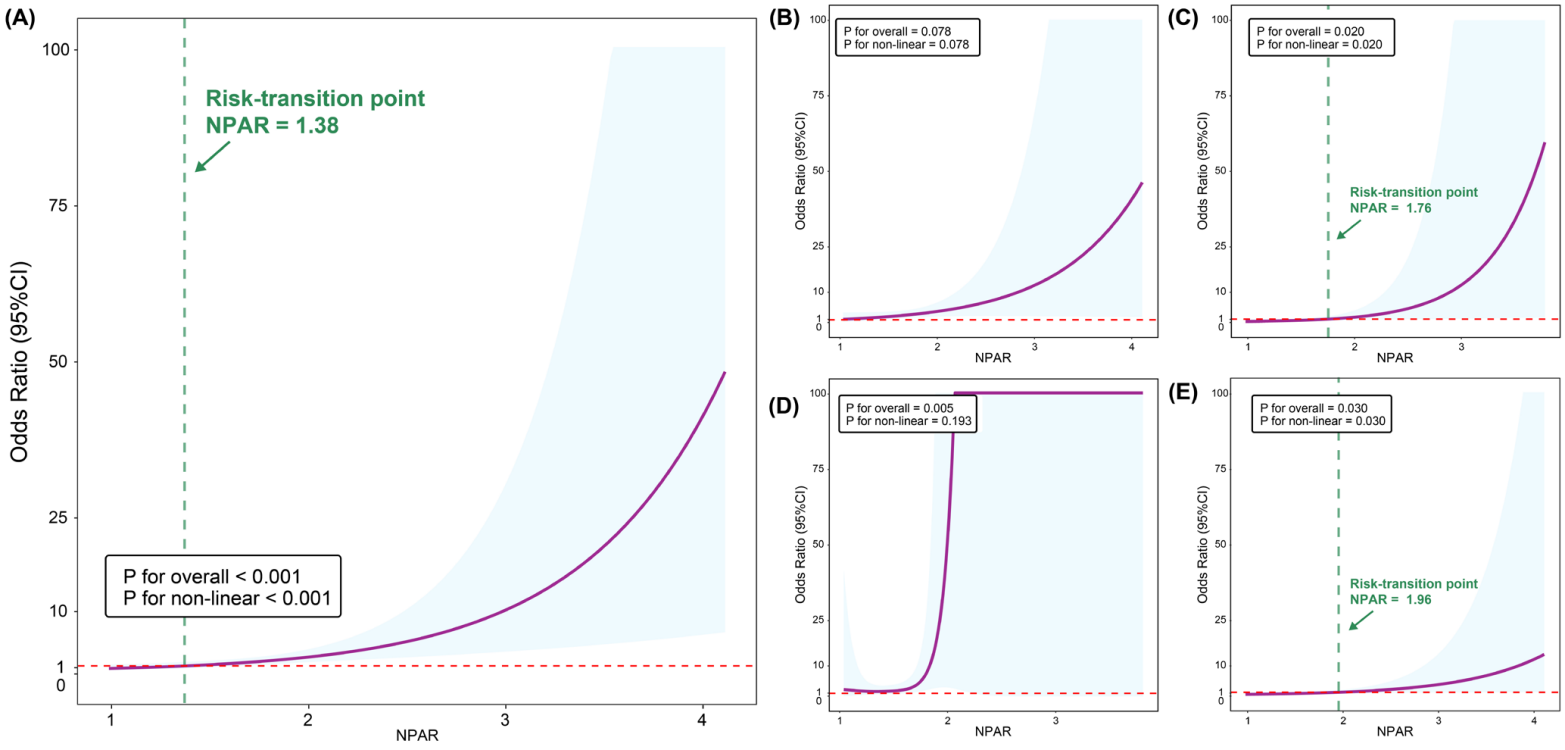
Dose–response relationship between NPAR and 2‐year progression of DKD. (A) DKD progression in all participants; (B) DKD progression in participants with eGFR above 60 mL/min/1.73 m^2^; (C) DKD progression in participants with eGFR below 60 mL/min/1.73 m^2^; (D) DKD progression in participants aged less than 60 years; (E) DKD progression in participants aged 60 years or older. DKD, diabetic kidney disease; eGFR, estimated glomerular filtration rate; NPAR, neutrophil percentage‐to‐albumin ratio.

As shown in Figure 2A, RCS analysis revealed a significant nonlinear association between NPAR and the 2‐year composite kidney outcome in patients with diabetic nephropathy (*p* < 0.001; *p* for non‐linear < 0.001). The progression risk increased with rising NPAR levels, with a more pronounced increase beyond the threshold of 1.38. Subgroup analyses further delineated distinct patterns of association (Figure 2B–E). A nonlinear relationship was observed in patients with eGFR < 60 mL/min/1.73 m^2^ (*p* for non‐linear = 0.020, threshold = 1.76; Figure 2C) and in patients aged ≥ 60 years (*p* for non‐linear = 0.030, threshold = 1.96; Figure 2E). In contrast, a linear positive association was found in patients with eGFR ≥ 60 mL/min/1.73 m^2^ (*p* for non‐linear = 0.078; Figure 2B) and in those aged < 60 years (*p* for non‐linear = 0.193; Figure 2D).

### 3.5 Establishment of Prognostic Model

**FIGURE 3:**
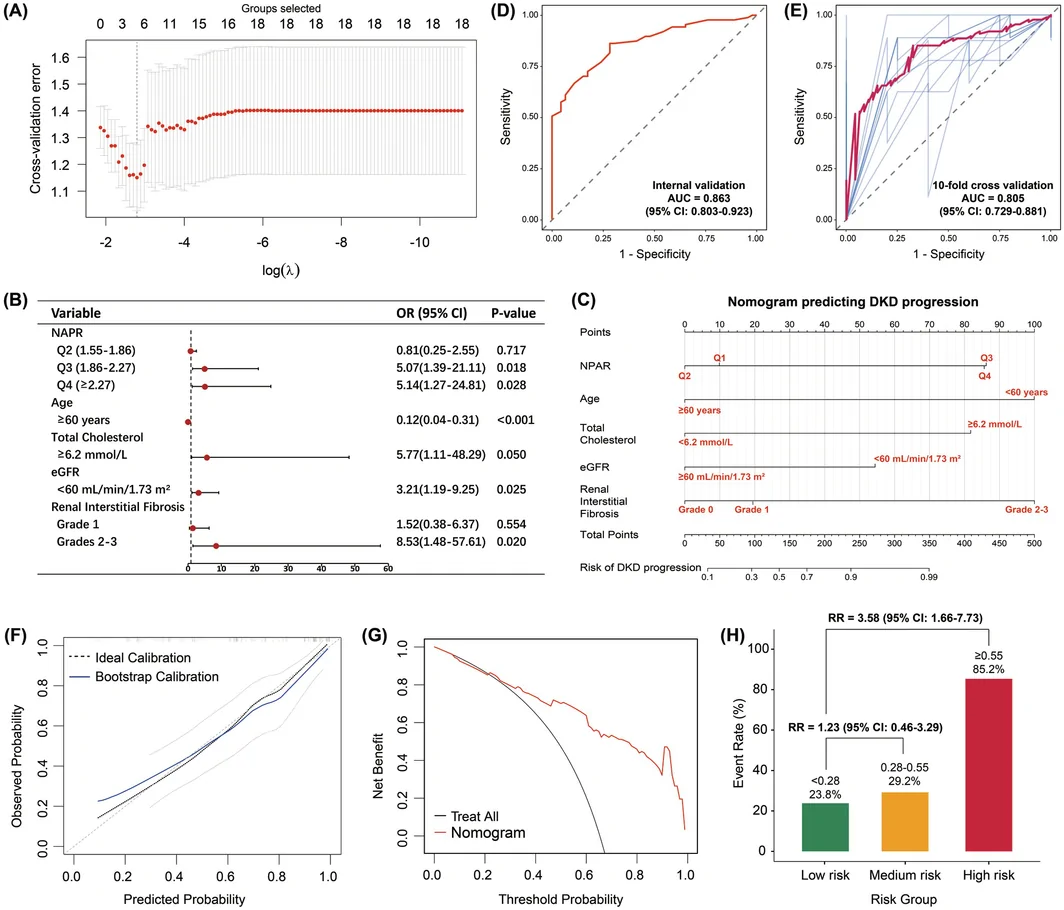
The prognostic model for predicting 2‐year progression of diabetic kidney disease. (A) Group selection using grMCP coefficients profiles (*y*‐axis) of features; (B) Forest plot showing the results of multivariable Logistic analysis; (C) Nomogram predicting DKD progression; (D) Internal validation AUROC curve; (E) 10‐fold cross‐validated AUROC curve; (F) Calibration curve of the model; (G) The decision curve of model; (H) Risk stratification based on Youden index. AUROC, area under the receiver operating characteristic curve; CI, confidence interval; DKD, diabetic kidney disease; eGFR, estimated glomerular filtration rate; grMCP, group minimax concave penalty; NPAR, neutrophil percentage‐to‐albumin ratio; RR, relative risk.

We further applied group selection using grMCP to select five candidate predictors with non‐zero coefficients, including NPAR quartiles, age, total cholesterol, eGFR, and renal interstitial fibrosis (Figure 3A). Then these variables were incorporated into a multivariable logistic regression model to quantify their associations with the renal outcome, yielding ORs ranging from 0.12 to 8.53 (Figure 3B). A nomogram was subsequently constructed based on this logistic model to predict the 2‐year composite renal outcome, and the AUROC was 0.863 (95% CI: 0.803–0.923) (Figure 3C,D). To assess robustness and internal validity, we performed 10‐fold cross‐validation. The AUROC decreased from 0.863 in the training set to 0.805 in cross‐validation, while remaining indicative of good discriminative performance (Figure 3E). The calibration curves indicated close agreement between predicted and observed probabilities, and decision curve analysis supported favorable clinical utility (Figure 3F,G). Furthermore, patients were stratified into low‐, medium‐, and high‐risk groups based on cut‐off values of half the Youden index (0.28) and the Youden index itself (0.55). The progression rates in these groups were 23.8%, 29.2%, and 85.2%, respectively. Compared to the low‐risk group, the high‐risk group had a relative risk (RR) of progression of 3.58 (95% CI: 1.66–7.73) (Figure 3H).

### 3.6 Sensitivity Analyses

Given the substantial proportion of DKD patients lost to follow‐up, we conducted a sensitivity analysis comparing baseline demographic, clinical, renal pathological, and therapeutic characteristics between those who completed follow‐up and those lost to follow‐up. No statistically significant differences were observed between the two groups (Tables S3 and S4). To address potential confounding, we applied inverse probability of treatment weighting (IPTW). In the IPTW‐weighted sample, DKD patients in NPAR quartile 4 (Q4) exhibited the highest risk of 2‐year DKD progression relative to those in quartile 1 (Q1), with an adjusted odds ratio (OR) of 7.03 (95% CI: 1.26–39.26). When modeled as an ordinal variable, higher NPAR quartiles were significantly associated with increased odds of DKD progression (adjusted OR per quartile increase = 2.06; 95% CI: 1.18–3.61; Table S5).

### 3.7 Association of Renal MPO Expression With NPAR and Histopathological Injury in DKD

**FIGURE 4:**
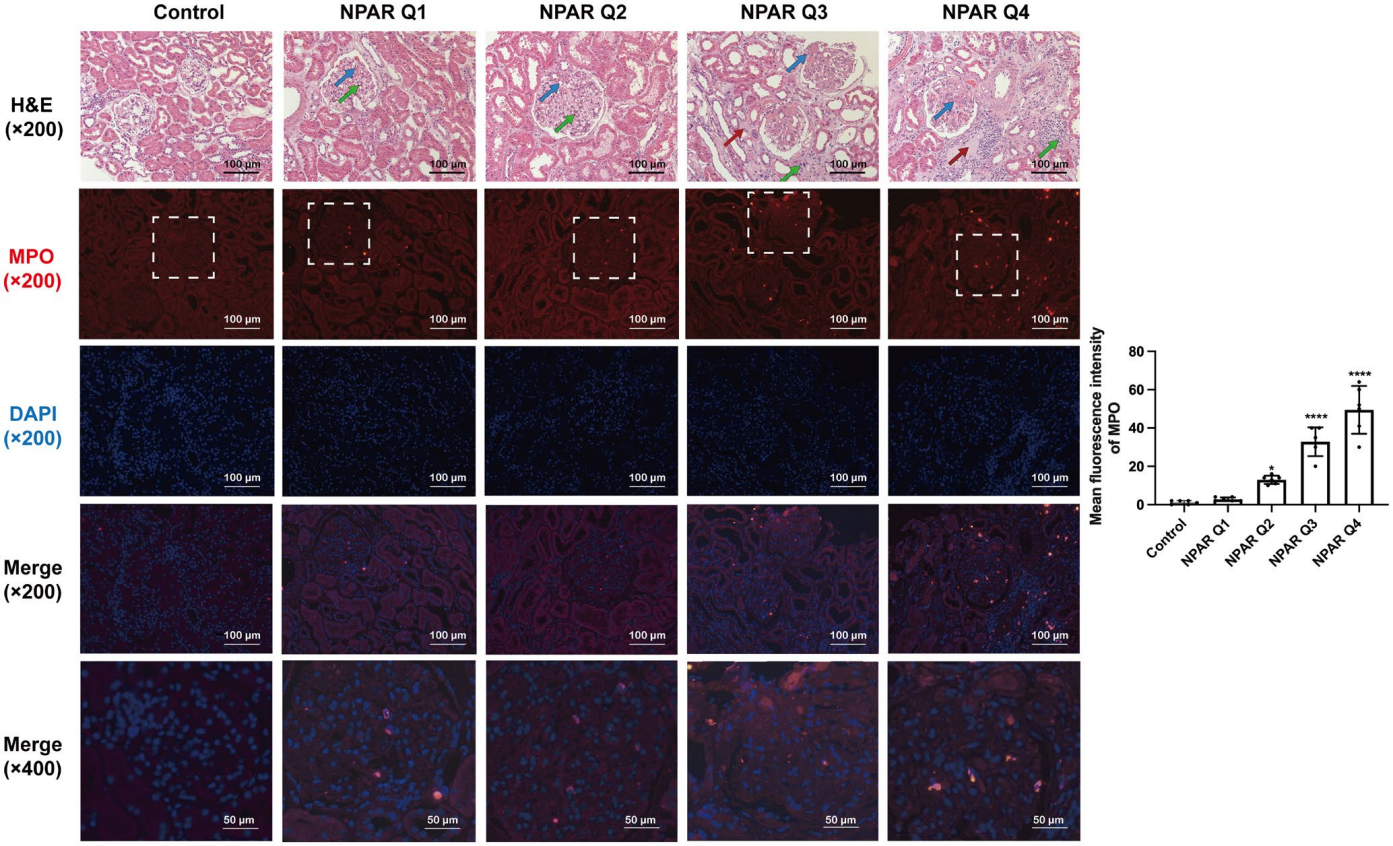
Increased renal MPO immunofluorescence reflects greater neutrophil accumulation across NPAR quartiles in DKD. Representative H&E and MPO immunofluorescence (IF) images (original magnification, ×200) of renal biopsy specimens from patients with diabetic kidney disease (DKD) stratified according to NPAR quartiles are shown. Representative higher‐magnification inset images (original magnification, ×400) are provided to facilitate visualization of MPO‐positive neutrophils. Blue arrows indicate mesangial expansion; red arrows indicate tubulointerstitial fibrosis and tubular atrophy (IFTA); and green arrows indicate interstitial inflammatory cell infiltration. Quantitative analysis of MPO mean fluorescence intensity (MFI), measured using ImageJ software, is shown for each group. Data are presented as mean ± SEM (*n* = 6 per group). Scale bars = 100 μm for the main panels and 50 μm for the inset images. *p < 0.05 and ****p < 0.0001 versus the NPAR Q1 group. H&E, hematoxylin and eosin; IF, immunofluorescence; MPO, myeloperoxidase; NPAR, neutrophil percentage‐to‐albumin ratio.

To investigate the contribution of neutrophils to DKD pathogenesis, we performed MPO immunostaining in renal biopsy specimens from patients with DKD stratified by NPAR quartiles and quantified neutrophil infiltration within glomerular and tubulointerstitial compartments. MPO staining was markedly increased in patients with higher NPAR quartiles compared with those in lower quartiles (Figure 4). Moreover, renal MPO expression positively correlated with fractional mesangial area, interstitial fibrosis, and tubular atrophy. Collectively, these findings support an association between renal neutrophil accumulation and glomerular and tubulointerstitial injury during DKD progression.

## 4 Discussion

In this retrospective cohort of 305 patients with biopsy‐confirmed DKD, we demonstrated that elevated NPAR was independently associated with more severe clinicopathological injury and an increased risk of renal progression. Besides, we developed a novel prognostic nomogram by modeling five predictive variables (NPAR quartiles, age, total cholesterol, eGFR and renal interstitial fibrosis) to predict the renal outcome for patients with DKD. This model showed excellent discrimination with an AUROC of 0.863 (95% CI: 0.809–0.926) for predicting adverse renal outcomes. These findings suggest that NPAR may serve as a useful biomarker for risk stratification in patients with DKD.

NPAR integrates information related to both inflammation and disease severity. In light of the growing recognition of inflammation as a key driver of DKD progression, the observed association between NPAR and adverse renal outcomes may, at least in part, reflect underlying inflammatory mechanisms. Growing evidence supports a central role for inflammation in the pathogenesis and progression of DKD [17, 18]. Hyperglycemia, advanced glycation end products, and oxidative stress activate innate immune pathways and promote the recruitment of inflammatory cells into the kidney [19]. Among these immune populations, neutrophils have increasingly been recognized as active participants in diabetic renal injury rather than passive bystanders. Activated neutrophils release proteases, reactive oxygen species, and pro‐inflammatory mediators that contribute to endothelial dysfunction, mesangial expansion, and tubulointerstitial fibrosis [20]. In addition, neutrophil extracellular traps (NETs) have been implicated in diabetic kidney injury through amplification of inflammatory signaling and tissue damage [21, 22, 23]. In DKD, albuminuria is strongly associated with biomarkers of inflammation, causing endothelial dysfunction and oxidative damage. It is widely acknowledged that albuminuria is an early clinical indicator of DKD, and its severity parallels DKD progression [24]. A prospective observational cohort study conducted by Gupta et al. demonstrated that CKD participants with rapid loss of kidney function and higher albuminuria exhibited elevated plasma levels of pro‐inflammatory cytokines [25]. These findings support the close interplay between albuminuria and systemic inflammation during DKD progression. Collectively, these observations support the concept that neutrophil‐mediated inflammation may contribute to the progression of DKD.

To further explore the biological relevance of NPAR at the tissue level, we performed MPO immunostaining and quantified neutrophil infiltration in renal biopsy specimens from patients with DKD stratified according to NPAR quartiles. We observed increased renal MPO expression and greater neutrophil accumulation in patients with higher NPAR quartiles. These findings provide tissue‐level evidence linking elevated NPAR to renal inflammatory cell accumulation and histopathological injury. Previous single‐cell RNA‐sequencing studies have revealed dynamic activation of immune and inflammatory signaling pathways during DKD progression, supporting a role for leukocyte recruitment in diabetic renal injury [26]. Our observations extend these findings by suggesting that neutrophil accumulation may represent one component of the inflammatory microenvironment associated with progressive DKD. The observed association between renal MPO expression and mesangial expansion, interstitial fibrosis, and tubular atrophy further supports a link between neutrophil accumulation and structural kidney injury.

Nevertheless, the present study does not establish a causal relationship between neutrophil infiltration and renal injury, nor does it directly assess neutrophil effector functions such as NETosis, degranulation, cytokine production, or reactive oxygen species generation. Therefore, the proposed contribution of neutrophils to DKD progression should be considered hypothesis‐generating rather than mechanistically proven. Future studies incorporating multiplex immunofluorescence, spatial transcriptomics, and functional assessment of neutrophil activation will be required to define the precise role of neutrophils in diabetic kidney injury.

Several epidemiological studies have reported positive associations between NPAR and the prevalence of DKD in patients with diabetes [12, 27, 28]. However, these investigations primarily focused on disease prevalence and lacked histopathological confirmation. In contrast, our study was conducted in a biopsy‐confirmed DKD cohort and demonstrates that elevated NPAR is independently associated with long‐term renal progression. Importantly, the association between NPAR and pathological severity suggests that this biomarker reflects not only systemic inflammation but also ongoing renal injury at the tissue level.

The biological interpretation of NPAR warrants careful consideration. NPAR integrates two readily available clinical parameters: neutrophil percentage, a marker of systemic innate immune activation, and serum albumin, which is influenced by both inflammatory burden and proteinuric kidney injury. Because hypoalbuminemia in DKD may arise from urinary protein loss, systemic inflammation, malnutrition, or a combination of these factors, the prognostic value of NPAR likely reflects the combined effects of inflammation, nutritional status, and kidney injury rather than a purely inflammatory signal. Previous studies have demonstrated that increased neutrophil counts and reduced serum albumin levels are each independently associated with DKD progression. Therefore, NPAR should be interpreted as an integrated biomarker of disease severity that captures multiple pathophysiological processes relevant to DKD progression [9, 29]. Importantly, the association between NPAR and adverse renal outcomes remained significant after adjustment for established clinical and pathological indicators of disease severity, suggesting that NPAR provides prognostic information beyond that conveyed by serum albumin alone and may better capture the combined effects of inflammation, nutritional status, and kidney injury that characterize progressive DKD.

A major strength of this study is the use of a biopsy‐confirmed DKD cohort with long‐term follow‐up. Histopathological confirmation enabled us to examine the relationship between NPAR, renal injury, and clinical outcomes at both the tissue and patient levels. From a clinical perspective, NPAR is derived from routinely available laboratory parameters and may represent a practical and inexpensive biomarker for risk stratification. Because it requires only neutrophil percentage and serum albumin, which are routinely measured during the clinical evaluation and follow‐up of patients with DKD, NPAR could be readily incorporated into routine clinical practice without additional cost or testing. Patients with elevated baseline NPAR may benefit from closer monitoring of renal function and proteinuria, as well as timely optimization of renoprotective therapies. Furthermore, incorporation of NPAR into our prognostic model improved individualized risk assessment.

Several limitations should be acknowledged. First, this was a single‐center retrospective study, which may be subject to selection bias and residual confounding. Although the prognostic model demonstrated good performance in the present cohort, prospective validation in independent external cohorts is required to confirm its generalizability and clinical utility before routine clinical implementation. Second, patients with missing data or incomplete follow‐up were excluded, which may have introduced selection bias. Third, NPAR was assessed at a single time point, and longitudinal changes in NPAR were not evaluated. Future studies are warranted to determine whether dynamic changes in NPAR provide additional prognostic information. Fourth, the observed interaction between SGLT2 inhibitor (SGLT2i) use and NPAR should be interpreted with caution because of the relatively small number of SGLT2i users and the observational, non‐randomized nature of treatment allocation. Therefore, this finding should be considered hypothesis‐generating rather than confirmatory and requires validation in larger prospective studies. Fifth, although MPO immunofluorescence demonstrated increased neutrophil accumulation in the kidneys of patients with higher NPAR quartiles, it primarily reflects neutrophil presence rather than functional activation. NET‐specific markers, such as citrullinated histone H3 and MPO–DNA complexes, as well as other neutrophil functional phenotypes, including degranulation, cytokine production, and reactive oxygen species generation, were not evaluated. Future studies integrating NET‐specific markers and functional analyses of neutrophil activation will be important to further elucidate the mechanistic contribution of neutrophils to DKD progression. In addition, the lower use of ACEI/ARB therapy in patients with higher NPAR quartiles may have reflected treatment contraindications associated with advanced kidney disease. Although baseline kidney function was adjusted for in the multivariable analyses, information regarding specific contraindications, treatment discontinuation, or adverse events was unavailable, and residual confounding cannot be excluded.

In conclusion, elevated NPAR was associated with more severe histopathological injury and an increased risk of renal progression in patients with biopsy‐confirmed DKD. As a simple and readily available biomarker, NPAR may provide incremental value for risk stratification and prognostic assessment and may reflect the inflammatory and injury‐related processes underlying DKD progression.

## Author Contributions

Yan Dai and Xiaoqiang Ding provided the original idea and designed the study, Hongyu Wang and Wendi He conducted data collection, Yang Li, Nana Song, Jie Li conducted the statistical analysis, Yi Fang, Yan Dai drafted the manuscript, Shi Jin, Hong Liu performed pathologic scoring of renal biopsy specimens. All authors contributed to the discussion and reviewed and edited the manuscript. Yang Li and Yan Dai are the guarantors of this work, as such, had full access to all the data in the study and took responsibility for the integrity of the data and the accuracy of the data analysis. All authors contributed to the article and approved the submitted manuscript.

## Funding

This research was funded by the National Key Research and Development Program of China (Grant No. 2024YFC3607400); the National Natural Science Foundation of China (Grant No. 82570848); the Innovation Plan of Shanghai Science and Technology Commission (Grant No. 25SF1905500); the Natural Science Foundation of Shanghai Municipality (Grant No. 22ZR1410600); the Shanghai Clinical Research Center (Grant No. 22MC1940100); the Shanghai Municipal Health Commission (Grant No. 20224Y0284).

## Ethics Statement

The study was conducted in accordance with the Declaration of Helsinki and approved by the Clinical Research Ethics Committee of Zhongshan Hospital (Approval No.: B2023‐076R).

## Conflicts of Interest

The authors declare no conflicts of interest.

## Supporting information


**Table S1:** Baseline demographic and clinical characteristics associated with the progression of diabetic nephropathy.
**Table S2:** Baseline renal pathological and therapeutic characteristics associated with the progression of diabetic nephropathy.
**Table S3:** Comparison of baseline demographic and clinical characteristics between DKD patients with and without follow‐up.
**Table S4:** Comparison of baseline renal pathological and therapeutic characteristics between DKD patients with and without follow‐up.
**Table S5:** Association between NPAR and the 2‐year progression of DKD by using inverse probability of treatment weighting (IPTW).

## Data Availability

Data supporting the findings of this study are available from the corresponding author upon reasonable request.
