# Supplementary material for: Association of Neutrophil Percentage‐to‐Albumin Ratio (NPAR) With Pathological Features and Long‐Term Prognosis in Diabetic Kidney Disease: A Cohort Study
Source: FASEB J. 2026 Sep 25;40(19):e72253. doi: 10.1096/fj.202601835RRR (PMC13614428; doi:10.1096/fj.202601835RRR)
Supplement: Supplementary file 1 [file FSB2-40-e72253-s001.pdf]

Supplementary Table 1. Baseline demographic and clinical characteristics associated with the progression of diabetic nephropathy

Variables	DKD progression within two years		Statistics	P-value
	Yes	No		
Age, years	52.55±12.10	59.27±11.05	-3.320	0.001[a]
Male, n (%)	78(84.8)	41(83.7)	0.030	0.863
BMI, kg/m²	25.91±3.88	24.66±3.56	1.922	0.057[a]
Smoking, n (%)	29(31.5)	18(36.7)	0.391	0.532[b]
Alcohol use, n (%)	13(14.1)	14(28.6)	4.307	0.038[b]
Hypertension, n (%)	92(100.0)	43(87.8)	11.766	<0.001[b]
Diabetes type, n (%)			0.063	0.803[b]
T1DM	3(3.3)	2(4.1)		
T2DM	89(96.7)	47(95.9)		
Other types	0(0)	0(0)		
Diabetes duration, years	10.83±6.45	12.16±6.97	0.236	0.871[a]
Hb, g/L	105.80±18.80	120.98±22.32	-4.055	<0.001[a]
NC, ×10⁹/L	4.21±1.43	4.50±3.93	-0.502	0.618[a]
NP, %	62.82±9.12	60.62±9.76	1.304	0.196[a]
LC, ×10⁹/L	1.70±0.82	1.74±0.58	-0.389	0.698[a]
MC, ×10⁹/L	0.56±0.47	0.53±0.23	0.518	0.605[a]
TP, g/L	57.47±8.07	62.63±8.29	-3.555	<0.001[a]
Alb, g/L	32.64±6.34	37.27±5.29	-4.609	<0.001[a]
BUN, mmol/L	11.1[8.5, 15.6]	8.6[6.9, 10.5]	3057.5	<0.001[c]
SUA, µmol/L	390[338, 453]	404[359, 467]	2081.5	0.580[c]
SCr, µmol/L	172[128, 226]	115[96, 146]	3269.5	<0.001[c]
eGFR, mL/min/1.73 m²	37[24, 57]	60[42, 74]	1314.5	<0.001[c]
Glu, mmol/L	6.84±2.71	7.42±3.71	-0.945	0.348[a]
HbA1c, %	7.1[6.3, 8.7]	7.0[6.2, 8.8]	2017.0	0.532[c]
TC, mmol/L	4.85±1.58	4.31±1.03	2.411	0.017[a]
TG, mmol/L	2.04±2.40	2.34±1.94	-0.796	0.428[a]
LDL-C, mmol/L	2.82±1.25	2.37±0.87	2.461	0.015[a]
HDL-C, mmol/L	1.11±0.41	1.01±0.28	1.732	0.086[a]
CRP, mg/L	1.20[0.50, 3.28]	1.30[0.70, 3.50]	2135	0.757[c]
ESR, mm/h	39.0[22.5, 55.0]	19.5[9.0, 46.0]	2121.5	0.002[c]
PCT, ng/mL	0.09[0.05, 0.11]	0.06[0.04, 0.09]	2196.0	0.008[c]
24-h UTP, g/24 h	4.62[2.82, 7.04]	1.24[0.61, 3.01]	3585.5	<0.001[c]
UACR, µg/mg Cr	3312[2096, 4943]	787[333, 2021]	3520.0	<0.001[c]
uIgG, mg/L	235.5[126.8, 355.5]	56.2[16.1, 124.5]	2857.0	<0.001[c]
uβ2-MG, mg/L	2.66[0.43, 11.93]	1.62[0.46, 4.20]	2108.5	0.097[c]

uα1-MG, mg/L	30.3[18.0, 49.5]	19.1[12.8, 28.6]	2419.5	<0.001[c]
uTRF, mg/L	99.17[56.1, 153.5]	33.9[7.3, 67.0]	2745.0	<0.001[c]

a Student's t-test; b Pearson chi-square test; c Wilcoxon rank-sum test.

Abbreviations: Alb, albumin; BMI, body mass index; BUN, blood urea nitrogen; CRP, C-reactive protein; DKD, diabetic kidney disease; eGFR, estimated glomerular filtration rate; ESR, erythrocyte sedimentation rate; Glu, glucose; Hb, hemoglobin; HbA1c, glycated hemoglobin; HDL-C, high-density lipoprotein cholesterol; LC, lymphocyte count; LDL-C, low-density lipoprotein cholesterol; MC, monocyte count; NC, neutrophil count; NP, neutrophil percentage; PCT, procalcitonin; SCr, serum creatinine; SUA, serum uric acid; T1DM, type 1 diabetes mellitus; T2DM, type 2 diabetes mellitus; TC, total cholesterol; TG, triglycerides; TP, total protein; UACR, urinary albumin-to-creatinine ratio; uIgG, urinary immunoglobulin G; uTRF, urinary transferrin; uα1-MG, urinary α1-microglobulin; uβ2-MG, urinary β2-microglobulin; 24-h UTP, 24-hour urinary protein.

Supplementary Table 2. Baseline renal pathological and therapeutic characteristics associated with the progression of diabetic nephropathy

Variables	DN progression within two years		Statistics	P-value
	Yes	No		
Diabetic nephropathy class, n (%)			12.936	<0.001[a]
Class 1-2	11(12.0)	16(32.7)		
Class 3	44(47.8)	25(51.0)		
Class 4	37(40.2)	8(16.3)		
Glomerular lesion grade, n (%)			11.211	<0.001[a]
Grades 1–3	1(1.0)	3(6.1)		
Grade 4	55(59.8)	39(79.6)		
Grade 5	36(39.1)	7(14.3)		
K–W nodule, n (%)	75(81.5)	28(57.1)	9.652	0.002[b]
Exudative lesion, n (%)	34(37.0)	6(12.2)	9.608	0.002[b]
Global glomerulosclerosis, n (%)			9.610	0.008[a]
Grade 1	34(37.0)	25(51.0)		
Grade 2	24(26.1)	18(36.7)		
Grade 3	34(37.0)	6(12.2)		
Renal interstitial fibrosis, n (%)			15.523	<0.001[a]
Grade 0	6(6.5)	9(18.4)		
Grade 1	52(56.5)	36(73.5)		
Grades 2–3	34(37.0)	4(8.2)		
Renal tubular atrophy, n (%)			17.238	<0.001[a]
Grade 1	16(17.4)	21(42.9)		
Grade 2	34(37.0)	21(42.9)		
Grade 3	42(45.7)	7(14.3)		
Arteriolar hyalinosis, n (%)	91(98.9)	48(98.0)	0.218	0.648[b]
Lipid-lowering treatment, n (%)	59(64.1)	22(44.9)	4.838	0.028[b]
SGLT2i treatment, n (%)			0.492	0.782[b]
After biopsy	32(65.3)	65(70.7)		
Before biopsy	12(24.5)	20(21.7)		
No	5(10.2)	7(7.6)		
ACEI/ARB treatment, n (%)	54(58.7)	40(81.6)	7.569	0.006[b]

a Cochran–Mantel–Haenszel (CMH) test; b Pearson chi-square test.

Abbreviations: ACEI, angiotensin-converting enzyme inhibitor; ARB, angiotensin receptor blocker; CI, confidence interval; DKD, diabetic kidney disease; K–W nodule, Kimmelstiel–Wilson nodule; SGLT2i, sodium-glucose cotransporter 2 inhibitor.

Supplementary Table 3. Comparison of baseline demographic and clinical characteristics between DKD patients with and without follow-up

Variables	Keeping follow-up at least 2 years		Statistics	P-value
	Yes (n=141)	No (n=157)		
Age, years	54.89±12.13	55.79±11.24	-0.444	0.657[a]
Male, n (%)	119(84.4)	122(77.7)	2.149	0.143[b]
BMI, kg/m²	25.48±3.81	24.94±3.29	1.295	0.197[a]
Smoking, n (%)	47(33.3)	52(33.1)	0.002	0.969[b]
Alcohol use, n (%)	27(19.1)	27(17.2)	0.191	0.662[b]
Hypertension, n (%)	135(95.7)	145(92.4)	1.502	0.220[b]
Diabetes type, n (%)			1.832	0.400[b]
T1DM	5(3.3)	5(3.2)		
T2DM	136(96.5)	150(95.5)		
Other types	0(0)	2(1.3)		
Diabetes duration, years	11.29±6.64	12.32±6.52	0.236	0.871[a]
Hb, g/L	111.08±21.29	110.32±23.45	0.291	0.772[a]
NC, ×10⁹/L	4.31±2.58	4.45±2.27	-0.513	0.608[a]
NP, %	62.06±9.37	63.12±9.73	-0.991	0.322[a]
LC, ×10⁹/L	1.71±0.74	1.71±0.65	0.039	0.969[a]
MC, ×10⁹/L	0.55±0.40	0.50±0.20	1.218	0.225[a]
TP, g/L	59.26±8.48	58.59±9.18	0.656	0.512[a]
Alb, g/L	34.25±6.37	33.41±7.21	1.064	0.288[a]
BUN, mmol/L	10.1[7.9, 13.4]	9.4[7.2, 13.0]	11916	0.210[c]
SUA, μmol/L	395[345, 464]	394[340, 447]	11490	0.499[c]
SCr, μmol/L	143[104, 199]	138[98, 194]	11694	0.341[c]
eGFR, mL/min/1.73 m²	46[30, 65]	47[31, 69]	10577	0.577[c]
Glu, mmol/L	7.05±3.10	6.90±3.59	0.389	0.697[a]
HbA1c, %	7.1[6.2, 8.7]	6.8[6.2, 8.4]	10240	0.400[c]
TC, mmol/L	4.66±1.43	4.99±1.95	-1.635	0.103[a]
TG, mmol/L	2.14±2.24	2.06±1.76	0.345	0.730[a]
LDL-C, mmol/L	2.67±1.15	3.10±2.21	-2.106	0.036[a]
HDL-C, mmol/L	1.07±0.37	1.14±0.45	-1.333	0.184[a]
CRP, mg/L	1.20[0.55, 3.40]	1.20[0.60, 3.90]	10316	0.660[c]
ESR, mm/h	32.0[16.0, 54.0]	36.0[16.5, 56.5]	7303.5	0.524[c]
PCT, ng/mL	0.07[0.05, 0.10]	0.07[0.05, 0.11]	9406.5	0.962[c]
24-h UTP, g/24 h	3.59[1.51, 6.02]	3.76[1.54, 6.09]	10523	0.583[c]
UACR, μg/mg Cr	2531[1054, 4262]	2862[1047, 4873]	9728	0.212[c]
uIgG, mg/L	152.5[55.5, 296.7]	149.3[60.2, 327.7]	9028	0.640[c]
uβ2-MG, mg/L	2.31[0.46, 7.14]	2.35[0.49, 6.93]	9548.5	0.820[c]

uα1-MG, mg/L	25.5[15.9, 41.2]	27.9[14.1, 49.0]	8995.5	0.541[c]
uTRF, mg/L	74.8[34.5, 132.9]	86.1[37.6, 133.7]	8823.5	0.444[c]

a Student's t-test; b Pearson chi-square test; c Wilcoxon rank-sum test.

Abbreviations: Alb, albumin; BMI, body mass index; BUN, blood urea nitrogen; CRP, C-reactive protein; DKD, diabetic kidney disease; eGFR, estimated glomerular filtration rate; ESR, erythrocyte sedimentation rate; Glu, glucose; Hb, hemoglobin; HbA1c, glycated hemoglobin; HDL-C, high-density lipoprotein cholesterol; LC, lymphocyte count; LDL-C, low-density lipoprotein cholesterol; MC, monocyte count; NC, neutrophil count; NP, neutrophil percentage; PCT, procalcitonin; SCr, serum creatinine; SUA, serum uric acid; T1DM, type 1 diabetes mellitus; T2DM, type 2 diabetes mellitus; TC, total cholesterol; TG, triglycerides; TP, total protein; UACR, urinary albumin-to-creatinine ratio; uIgG, urinary immunoglobulin G; uTRF, urinary transferrin; uα1-MG, urinary α1-microglobulin; uβ2-MG, urinary β2-microglobulin; 24-h UTP, 24-hour urinary protein.

Supplementary Table 4. Comparison of baseline renal pathological and therapeutic characteristics between DKD patients with and without follow-up

Variables	Keeping follow-up at least 2 years			P-value
	Yes (n=141)	No (n=157)		
Diabetic nephropathy class, n (%)			0.493	0.483[a]
Class 1-2	27(19.1)	26(16.6)		
Class 3	69(48.9)	76(48.4)		
Class 4	45(31.9)	55(35.0)		
Glomerular lesion grade, n (%)			2.318	0.314[a]
Grades 1–3	4(2.8)	9(5.7)		
Grade 4	94(66.7)	94(59.9)		
Grade 5	43(30.5)	54(34.4)		
K–W nodule, n (%)	103(73.0)	112(71.3)	0.108	0.742[b]
Exudative lesion, n (%)	40(28.4)	55(35.0)	1.519	0.218[b]
Global glomerulosclerosis, n (%)			0.273	0.873[a]
Grade 1	59(41.8)	66(42.0)		
Grade 2	42(29.8)	43(27.4)		
Grade 3	40(28.4)	48(30.6)		
Renal interstitial fibrosis, n (%)			0.766	0.682[a]
Grade 0	15(10.6)	14(8.9)		
Grade 1	88(62.4)	94(59.9)		
Grades 2–3	38(27.0)	49(31.2)		
Renal tubular atrophy, n (%)			0.375	0.829[a]
Grade 1	37(26.2)	46(29.3)		
Grade 2	55(39.0)	60(38.2)		
Grade 3	49(34.8)	51(32.5)		
Arteriolar hyalinosis, n (%)	139(98.6)	153(97.5)	0.480	0.488[b]
Lipid-lowering treatment, n (%)	81(57.4)	94(59.9)	0.180	0.671[b]
SGLT2i treatment, n (%)			0.388	0.824[b]
After biopsy	32(22.7)	31(19.7)		
Before biopsy	12(8.5)	14(8.9)		
No	97(68.8)	112(71.3)		
ACEI/ARB treatment, n (%)	94(66.7)	92(58.6)	2.061	0.151[b]

a Cochran–Mantel–Haenszel (CMH) test; b Pearson chi-square test.

Abbreviations: ACEI, angiotensin-converting enzyme inhibitor; ARB, angiotensin receptor blocker; CI, confidence interval; DKD, diabetic kidney disease; K–W nodule, Kimmelstiel–Wilson nodule; SGLT2i, sodium-glucose cotransporter 2 inhibitor.

Supplementary Table 5. Association between NPAR and the two-year progression of DKD by using inverse probability of treatment weighting (IPTW)

	**Univariable model OR (95% CI)**	**Multivariable model OR (95% CI)**
NPAR		
Q1	Reference	Reference
Q2	1.26(0.47-3.42)	1.81(0.39-8.29)
Q3	9.51(1.25-9.51)	6.59(1.14-38.24)
Q4	6.18(1.77-21.57)	7.03(1.26-39.26)
NPAR		
Ordinal variable	1.89(1.31-2.72)	2.06(1.18-3.61)

The multivariable regression model was adjusted for age, gender, BMI, hypertension history, serum uric acid, eGFR, fasting blood glucose, total cholesterol, CRP, diabetic nephropathy class, K-W nodule, renal interstitial fibrosis, renal tubular atrophy, lipid-lowering treatment, SGLT2i use, and ACEI/ARB use.

Abbreviations: ACEI, angiotensin-converting enzyme inhibitor; ARB, angiotensin receptor blocker; BMI, body mass index; CI, confidence interval; CRP, C-reactive protein; DKD, diabetic kidney disease; eGFR, estimated glomerular filtration rate; IPTW, inverse probability of treatment weighting; K-W nodule, Kimmelstiel−Wilson nodule; NPAR, neutrophil percentage-to-albumin ratio; OR, odds ratio; Q, quartile; SGLT2i, sodium-glucose cotransporter 2 inhibitor.
